# Cumulative childhood trauma and cybervictimization among Chinese college students: Internet addiction as a mediator and roommate relationships as a moderator

**DOI:** 10.3389/fpsyg.2022.791291

**Published:** 2022-08-17

**Authors:** Yunzi Xie, Jixia Wu, Chen Zhang, Lingyi Zhu

**Affiliations:** ^1^Department of Psychology, School of Education, Soochow University, Suzhou, China; ^2^School of Educational Sciences, Xuzhou University of Technology, Xuzhou, China; ^3^Graduate School of Education and Human Development, George Washington University, Washington, DC, United States

**Keywords:** cumulative childhood trauma, cybervictimization, internet addiction, roommate relationships, college students

## Abstract

Existing studies have found that childhood trauma is a risk predictor of cybervictimization, but few studies have explored the relationship between cumulative childhood trauma and college students’ cybervictimization. This study explored the relationship and the roles of Internet addiction and Internet victimization between them. A total of 854 college students (568 females, *M*_*age*_ = 18.92 years, *SD* = 0.86) completed a survey including the Short Form of Childhood Trauma Questionnaire, the Cyberbullying Inventory, the Young’s Internet Addiction Scale, and the revised Roommate Relationships Questionnaire. The results showed that: (1) cumulative childhood trauma was significantly positively associated with cybervictimization; (2) Internet addiction played a mediating role between cumulative childhood trauma and cybervictimization; and (3) roommate relationships played a moderating role between cumulative childhood trauma and cybervictimization, as well as Internet addiction and cybervictimization. The research findings provide a theoretical and practical basis for the prevention and intervention of college students’ cybervictimization.

## Introduction

According to “the 49th Statistical Report on China’s Internet Development,” published by the China Internet Network Information Center (2022), until December 2021, the number of Chinese Internet users reached 1.03 billion. The popularization of the Internet has brought a lot of convenience to society but has also created a series of problems, including cyberbullying. Cyberbullying refers to the repeated dissemination of hostile or offensive information through electronic or digital media by individuals or groups in an attempt to cause psychological injury or discomfort to others, and cybervictimization is the experience of being cyberbullied (Tokunaga, 2010). A recent cross-cultural study showed that the incidence of cybervictimization in China is as high as 44.5%, ranking fourth on the list (Zhu et al., 2021). The characteristics of cybervictimization, such as anonymity, freedom from time and space, a large number of potential audiences, long-term preservation of information, and fast dissemination speed, make its development much faster than traditional victimization (Kwak and Oh, 2017). At the same time, its harm to the victims should not be underestimated. Numerous studies have confirmed that cybervictimization can be a predictor of a series of problems among adults, such as depression, anxiety, loneliness, and suicidality, among others (Sargent et al., 2016; Wright, 2016; Cénat et al., 2019; Fang et al., 2020). College students have more autonomy when using the Internet, more discretionary time, and less academic pressure, which makes college campuses a hotbed of cybervictimization. Relevant research showed that 36.27% of Chinese college students reported being cybervictimized (Zhu et al., 2016). Therefore, it is necessary to explore the risk factors of cybervictimization among this population.

Childhood trauma refers to the consequences of behaviors that are actually or potentially harmful to the health, survival, growth, development, and dignity of the child by an individual who has the obligation to nurture, support, and supervise the child (Wang et al., 2019; Ma et al., 2020). Childhood trauma experience can adversely affect individuals’ short-term and long-term development (Stoltenborgh et al., 2015; Wang et al., 2019), and has also been shown to be a major risk factor for adults’ victimization (Desai et al., 2002; Ports et al., 2016). A long-term prospective study from childhood to middle age (40 years old) found that childhood abuse was significantly associated with an increased risk of lifetime revictimization, mainly in terms of interpersonal violence (Widom et al., 2008). Another meta-analysis also showed that childhood trauma is closely related to the victimization of intimate partner violence after adulthood (Li et al., 2019). People who have experienced childhood trauma often feel inferior, unlovable, helpless, and have low self-evaluation; these characteristics may weaken their ability to resist or recognize victimization (Cascardi, 2016), and lead them to repeat this cycle of victimization during college (Chapell et al., 2006). Cybervictimization, as an extension of traditional victimization, has received less attention in relation to childhood abuse experiences; few recent studies have shown a positive correlation between them (Watts et al., 2017). For example, Türk et al. (2021) found that childhood abuse experience is a predictor of college students’ cybervictimization, and that as individuals’ levels of childhood trauma increased, their cybervictimization also increased.

Cumulative childhood trauma refers to the accumulation of multiple types of trauma experienced by a child (Haselgruber et al., 2020). According to the Cumulative Risk Model of Polyvictimization (Finkelhor et al., 2007), victimization does not often occur in isolation, and individuals who have experienced multiple traumas are more likely to have persistent and severe psychological problems. Recent studies have demonstrated that the number of trauma types is a reliable predictor of mental health problems (Hodges et al., 2013; Choi and Oh, 2014; Hébert et al., 2018), and its influence exceeds the specific trauma type, duration, or frequency (Haselgruber et al., 2020). Multiple victimization experiences are also considered a risk factor for cybervictimization (Hamby et al., 2018). For instance, Chen et al. (2018) found that adolescents who experienced polyvictimization in the family were more likely to become internet victims. Another survey of 4,626 French college students showed that individuals who had experienced polyvictimization in childhood presented a higher prevalence of cybervictimization (Cénat et al., 2019). The spread of information and communication technologies has greatly increased the possibility of young people who have experienced multiple victimizations of being revictimized online (Hamby et al., 2018). In China, child trauma was not considered a social problem until the early 1990s (Wang et al., 2020). People’s awareness and concern about this issue have only gradually increased in recent years, and compared with today’s children and adolescents, college students with a history of childhood trauma are still a relatively neglected subgroup (Fu et al., 2018). The meta-analysis showed that the overall prevalence of childhood maltreatment among college students was 64.7% (Fu et al., 2018). Based on this, this study focused on Chinese college students to investigate the relationship between cumulative childhood trauma and cybervictimization.

### The mediating role of internet addiction

The Victim Schema Model (Rosen et al., 2007) proposes that individuals who have experienced victimization may develop a victim schema that leads to subsequent behavioral and emotional problems, which in turn are likely to lead to future victimization experiences. This shows that behavioral problems may play a role in the revictimization of victims. Internet addiction, also known as problematic Internet use, pathological Internet use, and compulsive Internet use (Widyanto and Griffiths, 2006), refers to excessive and uncontrollable use of the Internet (Young, 2004). As a problematic behavior related to Internet use and accompanied by negative emotions (Lozano-Blasco et al., 2022), whether Internet addiction plays a role in the relationship between cumulative childhood trauma and cybervictimization remains to be explored.

First, adverse childhood experiences are considered to be one of the most powerful risk predictors of addiction (Forster et al., 2018, 2021), and the newly revised I-PACE model (Brand et al., 2019) included childhood experience as the influencing variable of addictive behavior. Numerous studies have demonstrated that factors such as childhood traumatic experiences and parental psychological abuse can significantly and positively predict adults’ Internet addiction (Dalbudak et al., 2014; Arslan, 2017). For example, Yates et al. (2012) found through a survey of 1,470 college students that childhood maltreatment had an impact on problematic Internet use in early adults through the mediation of alexithymia. According to the Compensatory Internet Use Theory (Kardefelt-Winther, 2014), when individuals’ basic psychological needs are not met, they will tend to use the network as a kind of compensation, similar to substance use (Hruska and Delahanty, 2012). Individuals suffering from childhood trauma often fail to receive timely and appropriate responses and satisfaction for their physical and psychological needs in the long term, which motivates them to find alternative ways to satisfy themselves (Chen et al., 2021). The Internet, as an open and diverse world, provides them with a convenient and suitable platform to socially interact and communicate to alleviate the negative consequences of trauma or acquire relational satisfaction (Shevlin et al., 2015). This may lead to overuse and dependence on the Internet, which in turn increases the risk of Internet addiction.

Moreover, technology addiction, for example, mobile phone addiction (Herrero et al., 2021) and social media addiction (Çimke and Cerit, 2021), is significantly associated with cybervictimization. Internet addiction as a technology addiction is also considered one of the risk factors for cybervictimization (Boniel-Nissim and Sasson, 2018). Huang et al. (2021) found that the more time college students spend on the Internet, the more susceptible they are to be victimized in online games, and the higher the level of Internet addiction, the higher the risk of being victimized in social media. The results of another study also showed that Internet addiction can lead to an increase in sexual online victimization by lowering one’s physical self-esteem (Tamarit et al., 2021). In addition, Internet addiction emphasizes the extreme use of the Internet, which means that the individual needs to spend more time on the Internet, has strong negative emotions associated with withdrawal, and has negative effects on social life (Spada and Marino, 2017; Çevik et al., 2021). These factors also increase the risk of cybervictimization. Studies have shown that as the time and frequency of Internet use increases, young adults are more likely to be exposed to risks associated with the Internet, which further increases the risk of cybervictimization (Balakrishnan, 2015; Musharraf et al., 2019). Meanwhile, the adverse consequences of withdrawal associated with Internet addiction (e.g., fear of missing out) and the extreme preoccupation with the use of technological devices can also increase an individual’s vulnerability and potential risk of victimization in the online world (Craig et al., 2020; Varchetta et al., 2020). Finally, the obstruction of real social interactions by Internet addiction can also trigger individuals’ social incompetence, which in turn can lead to increased cybervictimization (Kostyunina et al., 2019). Based on the above analysis, we proposed the following hypothesis: Internet addiction mediates the relationship between cumulative childhood trauma and cybervictimization (H1).

### The moderating role of roommate relationships

Interpersonal relationships are considered critical protective factors for traditional and cyber victimization (Wang et al., 2009; Brighi et al., 2012; Livazovic and Ham, 2019; Martins et al., 2019). Moreover, individuals who have experienced childhood trauma are more likely to seek support and resources from relationships outside of their family (Healy and Sanders, 2018; Zhang et al., 2021). Exploring other effective interpersonal factors may provide certain social relationships that supplement college students with a history of cumulative childhood trauma in the prevention of and intervention for cybervictimization. University is an important period for the development of various interpersonal relationships (Chickering and Reisser, 1993), and good interpersonal relationships acquired during college can have a positive impact on individuals’ social adjustment and mental health, among others (Zhang and Zhao, 2021; Zhang et al., 2021). Studies have demonstrated that high levels of interpersonal relationships not only buffer the adverse effects of childhood abuse on adults (Cloitre et al., 2002; Collishaw et al., 2007) but also serves as a proximal protective factor for adults’ cybervictimization (Jenaro et al., 2018), and directly reduce the risk of cybervictimization among higher-education students (Heiman and Olenik Shemesh, 2019). Furthermore, good interpersonal relationships can also serve as social support for college students to provide emotional, technical, and tool support (Wills, 1985). According to the social support buffer hypothesis (Cohen and Wills, 1985), social support can buffer risk factors and their adverse consequences. Many studies have also confirmed that social support can not only reduce the risk of cybervictimization (Baldry et al., 2015; Herráiz and Gutiérrez, 2016; Elkady, 2019; Zych et al., 2019) but also plays a role in preventing revictimization of individuals who have experienced victimization (Pittenger et al., 2016; Hawn et al., 2018; Koçtürk and Bilge, 2018). Additionally, new supportive interpersonal relationships can also help individuals with addictive behaviors triggered by family dysfunction and trauma to regain social identity continuity or renew social identity (Dingle et al., 2015); this can alleviate the negative effects of addictive behaviors (McGaffin et al., 2017; Park et al., 2018; Zengİn and Naktİyok, 2022), reduce social isolation, and satisfy identity needs (Johansen et al., 2013; Dingle et al., 2015), further reducing cybervictimization.

Roommate relationships are a specific type of interpersonal relationship widely and uniquely experienced by college students (Erb et al., 2014). Compared with other campus peer relationships, roommates need to share physical space and communicate and get along for a long time. Such frequent contact and interaction provide the foundation for building and integrating intimate relationships (Whitmore, 2014), but also brings challenges (Erb et al., 2014). Some studies have found that high satisfaction with roommate relationships enhances college students’ sense of intimacy, campus belonging, emotional stability, and social capital (Shook and Clay, 2012; Omonijo et al., 2015; Yao, 2016). In addition, roommates can serve as capable guardians that provide social, emotional, and technical support to reduce offline victimization (Daigle et al., 2008) and online victimization (Milani et al., 2022), while roommate relationship problems were also demonstrated to increase the risk of cybervictimization (Crosslin and Crosslin, 2014).

In China, the dormitory is regarded as the most important micro-environmental system in college campus life and plays a key role in the formation and change of college students’ social networks (Ling and Kangli, 2021). Moreover, roommate relationships have become the most important type of interpersonal relationship for Chinese college students (Zhou et al., 2017). The vast majority of Chinese college students live in a uniform dormitory throughout their college years, and they spend more than half of their extracurricular time in the dormitory (Chen et al., 2012; Li et al., 2021). A study of students from the China Medical University found that the direct effect of roommate relationships on college students is much higher than that of other social factors (e.g., parenting style and professional satisfaction) (Lei et al., 2019). Especially in the context of the COVID-19 pandemic, the dormitory has become the main place for college students to study and live in the school, and the discussion of the role of roommate relationships has become more important and urgent. However, there is currently little empirical research on the role of roommate relationships in college students’ cybervictimization. As a type of interpersonal relationship, whether roommate relationships can also be a proximal influencing factor of cybervictimization, play a moderating role in the revictimization of college students who have experienced childhood abuse as well as the association between Internet addiction and cybervictimization, remains to be further discussed. Therefore, this study proposed a second hypothesis based on relevant research on interpersonal relationships and the social support buffering hypothesis: roommate relationships moderate the relationship between cumulative childhood trauma and cybervictimization (H2a), as well as Internet addiction and cybervictimization (H2b).

### The present study

**FIGURE 1:**
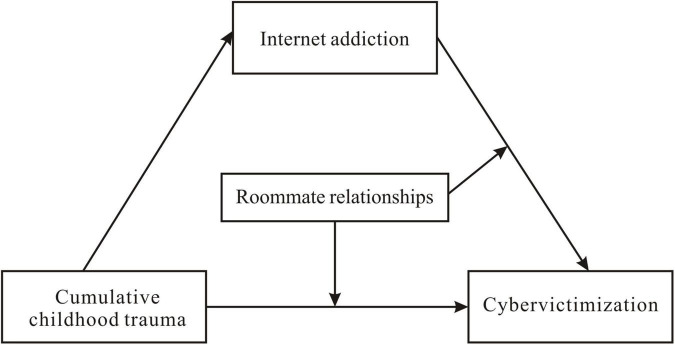
The hypothesized model of the present study.

This study was based on the Victim Schema Model (Rosen et al., 2007), the Cumulative Risk Model of Polyvictimization (Finkelhor et al., 2007), and the Social Support Buffer Hypothesis (Cohen and Wills, 1985), combined with existing research and using college students as a sample to explore the relationship between cumulative childhood trauma, Internet addiction, roommate relationships, and cybervictimization. Additionally, as previous research found gender differences in college students’ cybervictimization (Fang et al., 2020), we considered gender as a control variable in this study. The purpose of this research was to generate a moderated mediation model based on the combined effects described in Hypotheses 1 and 2 (see Figure 1).

## Materials and methods

### Participants

A total of 902 college students participated in the survey from two universities in Chongqing and Chengdu, China. First, referring to the screening method of Gerdner and Allgulander (2009), 32 participants were removed based on the validity items of the Childhood Trauma Questionnaire. Specifically, three validity items were included, when participants answered “5” on each item, it was coded as “1,” otherwise it was coded as “0,” and then the total score of the three items was calculated and participants with a total score of 3 were removed. Second, 16 participants were removed because they chose the same answer on all items in this survey. Finally, there were also 19 participants with 1 to 3 missing values; comparing the differences between these participants and the retained participants in each critical variable, the results showed no significant differences in cumulative childhood trauma (*t* = 0.52, *p* > 0.05), Internet addiction (*t* = 1.01, *p* > 0.05), roommate relationships (*t* = –1.93, *p* > 0.05), and cybervictimization (*t* = 0.75, *p* > 0.05), so the expectation maximization (EM) method was used to replace the missing value (Zhao, 2022). The final sample comprised 854 valid participants (568 females, aged 17–24, *M_*age*_* = 18.92, *SD* = 0.86), with a return rate of 94.68%. We used the G*Power application to test the sample size of 854 participants, 6 predictors, and an effect size of 0.15 as the baseline to further examine whether our valid sample was appropriate. The results of the *post hoc* power analysis revealed that at the 0.05 level, the power to detect the obtained effect for the whole regression in the prediction of cybervictimization was 0.89, which was above the value of 0.8 recommended by previous studies (Faul et al., 2009).

## Measures

### Cumulative childhood trauma

The Short Form of Childhood Trauma Questionnaire (CTQ-SF) (Bernstein et al., 2003) was used to assess the participants’ experience of childhood trauma. The scale has five subscales and a total of 28 items: emotional abuse (5 items; e.g., “I thought that my parents wished I had never been born.”), physical abuse (5 items; e.g., “People in my family hit me so hard that it left me with bruises or marks.”), sexual abuse (5 items; e.g., “Someone threatened me to engage in sexual behavior with them”), emotional neglect (5 reverse items; e.g., “People in my family felt close to each other.”), physical neglect (5 items; e.g., “My parents were too drunk or high to take care of the family”), and a validity scale (3 items; e.g., “I had the perfect childhood”). The scale uses a 5-point score (1 = never true, 5 = very often true), with higher scores representing higher levels of childhood trauma. Studies have shown that the Chinese version of the CTQ-SF has good reliability and validity among college students (He et al., 2019). The Cronbach’s α in this study was 0.86.

As for the calculation of the cumulative childhood trauma index, this study referred to the dichotomy proposed by Haselgruber et al. (2020). The total score for each subscale was first calculated, and then the cut-off for each subscale (Bernstein et al., 2003) was used to discriminate whether a certain type of childhood trauma had been experienced. The score of each subscale was coded as “1” when it reached the corresponding cut-off value, otherwise it was coded as “0.” By summing the five binary outcomes, a continuous cumulative trauma score ranging from 0 to 5 was calculated, indicating how many types of childhood trauma had been experienced.

### Cybervictimization

Cybervictimization was assessed using the Cyber Bullying Inventory (CBI), developed by Erdur-Baker and KavS̨ut (2007). There are 18 items in total, such as “Embarrassing photos of me were shot by a mobile phone without my permission.” Participants answered the questions on a 5-point scale (1 = never, 4 = more than five times). The higher the score, the greater the cyberbullying encounters. The CBI has been proven to have good reliability and validity among Chinese college students (Fang et al., 2020). The Cronbach’s α in this study was 0.82.

### Internet Addiction

Internet addiction was measured using the diagnostic scale compiled by Young (1998), which included eight items in total (e.g., “Do you feel preoccupied with the Internet?”). Participants made judgments based on statements (1 = no, 2 = yes). Individuals who answered “yes” to five or more of the criteria were classified as addictive Internet users. This scale has been proven to have good reliability and validity in Chinese college students (Mei et al., 2015), and the Cronbach’s α in this study was 0.68.

### Roommate relationships

The questionnaire was adapted from the peer relationship dimension in the Self-Description Questionnaire (SDQI) compiled by Marsh and Holmes (1990) and revised by Wang (2001). The original questionnaire has been proven to have good reliability and validity among Chinese college students (Wang and Xie, 2020). In this survey, the word “friend” was changed to “roommates,” for example, “I can get along well with my current roommates” and “my current roommates like me very much.” The scale has six items in total that are rated on a 5-point scale (1 = very inconsistent, 5 = very consistent). Higher scores reflect better roommate relationships. The Cronbach’s α of the modified questionnaire in this study was 0.88.

### Procedure

Participants signed up and completed the questionnaires on the *Wenjuanxing* platform (a popular Chinese survey website) in April 2021; it took approximately 20 min to complete the survey. Informed consent was obtained from all participants who were told that their participation was voluntary, anonymous, and confidential, and that they could withdraw from the study at any time. All materials and procedures used in the study were approved by the Ethical Committee for Scientific Research at Soochow University.

### Data analysis

SPSS 21.0 and PROCESS macro (Hayes, 2013) were used for statistical analysis. First, factor analysis was used to test for common method bias. Second, descriptive statistics and correlation analyzes were conducted for the correlation variables. Third, according to the model templates of Hayes (2013), Model 4 in the PROCESS macro was used to examine the mediating effect of Internet addiction. Fourth, Model 15 in the PROCESS macro was used to test the hypothetical moderating mediation model. All study variables were transformed using the proportion of maximum scaling (POMS) method, which will not modify the multivariate distribution and covariance matrix of the transformed variables (Little, 2013). The bootstrapping method produces 95% bias-corrected confidence intervals of these effects from 5,000 resamples of the data. Confidence intervals that did not contain zero indicated significant effects.

## Results

### Common variance bias

As all the data in this study came from self-reported questionnaires, the Harman single factor test was used to test the common method bias. The results showed that there were 15 factors with eigenvalues > 1, and the first factor explained 15.84% of the total variation, which was less than the critical value of 40% (Podsakoff et al., 2003), indicating that no serious common method bias existed in this study.

### Preliminary analyzes

Table 1 shows the descriptive statistics and correlation matrices of the study variables. The results showed that cumulative childhood trauma was positively correlated with Internet addiction and cybervictimization, and negatively correlated with roommate relationships. Internet addiction was negatively correlated with roommate relationships and positively correlated with cybervictimization. Roommate relationships were negatively correlated with cybervictimization.

**TABLE 1:** Descriptive statistics and correlations of main study variables.

	M	SD	1	2	3	4	5	6
1.Cumulative childhood trauma	0.27	0.68	1					
2.Internet addiction	1.10	0.16	0.15**	1				
3. Roommate relationships	3.54	0.50	−0.27**	−0.21**	1			
4.Cybervictimization	1.13	0.17	0.24**	0.20**	−0.20**	1		

**p < 0.01. Gender was dummy coded (1 = male, 0 = female).

### Mediating effect of internet addiction

Model 4 in the PROCESS macro was used to test the mediating effect of Internet addiction, and the results are shown in Table 2. After controlling for gender, cumulative childhood trauma was significantly associated with cybervictimization (β = 0.07, *p* < 0.001, CI = [0.05, 0.09]) and Internet addiction (β = 0.14, *p* < 0.001, CI = [0.08, 0.21]). In addition, Internet addiction was significantly associated with cybervictimization (β = 0.06, *p* < 0.001, CI = [0.04, 0.09]). The indirect effect of Internet addiction between cumulative childhood trauma and cybervictimization was 0.01 with a 95% confidence interval (CI) of [0.003, 0.02], and the mediating effect accounted for 12.50% of the total effect, which supports Hypothesis 1.

**TABLE 2:** The mediating effect of internet addiction.

Predictors	Model 1 (outcome: CV)			Model 2 (outcome: IA)			Model 3 (outcome: CV)		
	β	*SE*	*t*	β	*SE*	*t*	β	*SE*	*t*
CCT	0.07	0.01	6.37***	0.14	0.03	4.34***	0.07	0.01	6.37***
IA							0.06	0.01	5.59***
Gender	0.02	0.004	5.50***	–0.02	0.01	–1.93	0.02	0.004	5.94***
*R* ^2^	0.09			0.03			0.12		
*F*	41.00***			11.22***			38.73***		

CCT, cumulative childhood trauma; IA, Internet addiction; CV, cybervictimization. Gender was dummy coded (1 = male, 0 = female). ***p < 0.001.

### Moderated mediation effects

**FIGURE 2:**
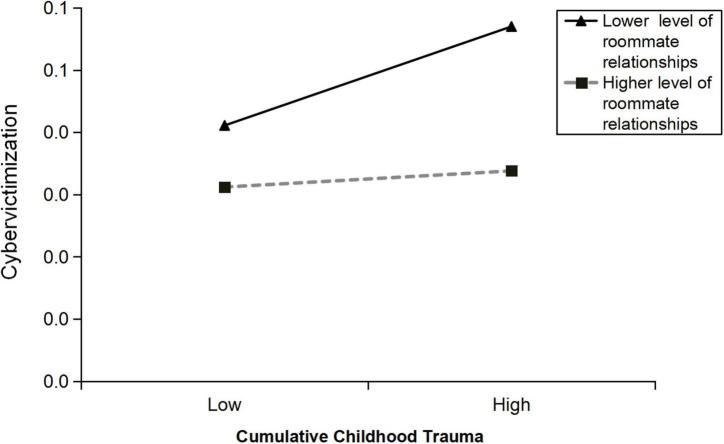
The simple slope analysis model shows that roommate relationships have a moderating effect between cumulative childhood trauma and cybervictimization.

Hypothesis 2 was tested using Model 15 in the PROCESS macro. As shown in Table 3, the interaction between cumulative childhood trauma and roommate relationships (CCT × RR) was significantly associated with cybervictimization (β = –0.22, *SE* = 0.10, CI = [–0.41, –0.02]), suggesting that roommate relationships played a moderating role between cumulative childhood trauma and cybervictimization. Simple slope analysis was used to better understand the moderating effect of roommate relationships; the results showed that when the score of roommate relationships was lower (i.e., 1 *SD* below the mean), cumulative childhood trauma was positively associated with cybervictimization (β_simple_ = 0.06, *SE* = 0.01, *p* < 0.001; see Figure 2); however when the roommate relationships score was higher (i.e., 1 *SD* above the mean), the relationship between cumulative childhood trauma and cybervictimization was not statistically significant (β_simple_ = 0.01, *SE* = 0.03, *p* = 0.68; see Figure 2).

**TABLE 3:** The moderated-mediating effect of cumulative childhood trauma on cybervictimization.

Predictors	Model 1 (outcome: IA)				Model 2 (outcome: CV)			
	β	SE	*t*	95%CI	β	SE	*t*	95%CI
CCT	0.14	0.03	4.35***	[0.08, 0.21]	0.18	0.06	3.19***	[0.07, 0.29]
CCT × RR					–0.22	0.10	–2.18*	[–0.41, –0.02]
IA					0.23	0.04	5.30***	[0.14, 0.31]
IA × RR					–0.29	0.07	–4.14***	[–0.43, –0.15]
Gender	–0.02	0.01	–1.91	[–0.05, 0.001]	0.02	0.004	5.82***	[0.02, 0.03]
R^2^	0.03				0.17			
*F*	11.22***				28.70***			

CCT, cumulative childhood trauma; IA, Internet addiction; CV, cybervictimization; RR, roommate relationships. Gender was dummy coded (1 = male, 0 = female). *p < 0.05, ***p < 0.001.

**FIGURE 3:**
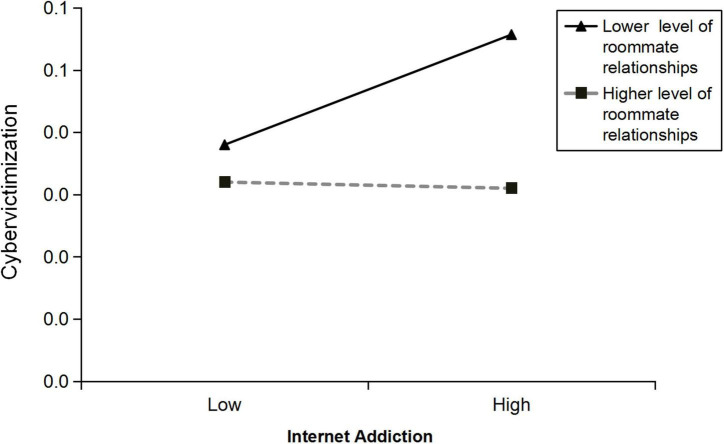
The simple slope analysis model shows that roommate relationships have a moderating effect between internet addiction and cybervictimization.

Moreover, the interaction between Internet addiction and roommate relationships (IA × RR) was significantly associated with cybervictimization (β = –0.29, *SE* = 0.07, *p* < 0.001, CI = [–0.43, –0.15]), suggesting that roommate relationships play a moderating role in the relationship between Internet addiction and cybervictimization. Simple slope analysis showed that, when the score of roommate relationships was lower (–1*SD*), the relationship between Internet addiction and cybervictimization was statistically significant (β_simple_ = 0.07, *SE* = 0.01, *p* < 0.001; see Figure 3), but when the score of roommate relationships was higher (+ 1*SD*), Internet addiction was not significantly associated with cybervictimization (β_simple_ = –0.004, *SE* = 0.02, *p* = 0.82; see Figure 3).

## Discussion

The Internet is an important platform for college students to interact, communicate, and explore themselves outside the real world. As a new type of victimization, cybervictimization has gradually become a public health issue of social concern (Cuadrado-Gordillo and Fernández-Antelo, 2020). Studies have confirmed the predictive effect of childhood trauma on cybervictimization among college students (Watts et al., 2017; Türk et al., 2021). However, the relationship between cumulative childhood trauma and cybervictimization among Chinese college students, and the role of Internet addiction and roommate relationships was yet to be further confirmed. In this study, a moderated mediation model for Chinese college students was constructed to explore the relationship between cumulative childhood trauma and cybervictimization. The results showed that cumulative childhood trauma was significantly positively correlated with cybervictimization, and the association was positively mediated by internet addiction. In addition, for individuals with lower roommate relationships, the association between cumulative childhood trauma and cybervictimization as well as Internet addiction and cybervictimization was significant.

### The mediating role of internet addiction

The present study confirmed the mediating role of Internet addiction between cumulative childhood trauma and cybervictimization, further validating the Victim Schema Model (Rosen et al., 2007) and its applicability in the context of online victimization; that is, college students who have multiple childhood abuse experiences are more likely to be revictimized in the online world, and problematic behavior (Internet addiction) mediates this relationship. Specifically, we found that cumulative childhood trauma was significantly positively associated with college students’ Internet addiction, which is consistent with previous studies (Yates et al., 2012; Dalbudak et al., 2014). According to the Basic Psychological Needs Theory (Ryan and Deci, 2000), satisfaction with psychological needs is the basic motivation for individual behavior. If the basic needs cannot be met, individuals will be forced to turn to other environments that can meet these needs. An adverse family environment makes it difficult for individuals to fully meet their physical and psychological needs; they may turn to the virtual world to avoid negative emotions or seek relational satisfaction, and the positive experiences generated in this process will activate their reward system (Longobardi et al., 2020) or make them regard the Internet as a common strategy to deal with pressure (Brand et al., 2019), which further leads to more frequent Internet use and ultimately results in Internet dependence or addiction (Song et al., 2004).

Conversely, Internet addiction was significantly positively associated with cybervictimization, that is, college students with higher levels of Internet addiction experience more cybervictimization. Increased time online is one of the basic characteristics of Internet addiction (Tokunaga, 2010), and features associated with the Internet such as anonymity and intertemporality make online aggression easier, making it more likely for individuals with Internet addiction to encounter negative users and evaluations, personal privacy exposure, and online stalking. A study showed that even users with more digital competence find it difficult to avoid the risk of cybervictimization (Staksrud et al., 2013). Second, according to the Person-Affect-Cognition-Execution (I-PACE) model for addictive behaviors (Brand et al., 2019), compensatory effects become stronger over gratifying effects in later stages of the addiction process. That is to say, with the development of Internet addiction behaviors, individuals need to continuously pursue stronger and newer stimuli to maintain the initial satisfaction, and these new and adventurous online activities are usually accompanied by more unknown dangers (e.g., visiting unfamiliar websites), which may also increase the risk of cybervictimization (Holfeld and Sukhawathanakul, 2017). Especially for individuals who have suffered childhood abuse, meeting social and emotional needs is one of their main motivations to surf the Internet, but an overemphasis and dependence on online relationships may also increase their vulnerability in the online world (Hoffner and Lee, 2015) while weakening their connection to reality (Cuadrado-Gordillo and Fernández-Antelo, 2020). This can lead to more (offline and online) interpersonal problems, which in turn reinforces the individual’s victim schema, trapping them in into the cycle of cybervictimization (Chu et al., 2019). This finding preliminarily verified the role of online problematic behaviors in childhood trauma and Internet victimization. Considering that Internet addiction is a relatively general concept, future research could also introduce other online problematic behaviors, such as social media addiction, for a more in-depth exploration.

### The moderating role of roommate relationships

Roommate relationship is an important and prevalent type of interpersonal relationship on college campuses in China. This study has explored whether roommate relationships can act as an interpersonal complement in the prevention of victims’ revictimization. The results showed that roommate relationships moderated the relationship between cumulative childhood trauma and cybervictimization, as well as Internet addiction and cybervictimization, but contrary to our expectation, the associations were significant only when college students who reported lower roommate relationships, which supports the reverse-buffering model (Rueger et al., 2016). This result is consistent with those found by Crosslin and Crosslin (2014). One possible explanation is that roommate relationships, as a proximal influencing factor of cybervictimization, play a positive role mainly through technology support and social interaction, among others, while childhood abuse experience and Internet addiction are both closely related to avoidance and negative problem coping styles (Lei et al., 2018; Liu et al., 2020), which reduces the likelihood of individuals actively seeking outside help and leads to further cybervictimization (Wright, 2015). In addition, cybervictimization is less likely to be detected than face-to-face victimization (Dennehy et al., 2020). Particularly for individuals who have experienced childhood trauma, their ability to recognize and prevent victimization is lower (Cascardi, 2016), making it difficult for roommate relationships to play a timely role in preventing cybervictimization.

Conversely, the supportive role of roommate relationships needs to be predicated on benign relationships, but according to the Interpersonal Theory (Liu and Kuo, 2007), parent-child relationships are closely correlated with adult interpersonal relationships. College students who have experienced childhood trauma may also extend patterns learned in the family to roommate relationships, directly affecting the benign development of these. Furthermore, Internet addiction is also thought to hinder individuals’ real-life interactions and increase interpersonal problems (Seo et al., 2009); therefore, roommate relationships are likely to be directly affected by childhood trauma and Internet addiction. In addition, cumulative childhood maltreatment implies that individuals have experienced more significant levels and more types of abuse, and Internet addiction is also an extreme form of problematic Internet use (Çevik et al., 2021), making the impact on cybervictimization too strong to be buffered by roommate relationships; instead, childhood trauma and cybervictimization can have an even stronger negative impact on roommate relationships, which in turn can increase the risk of cybervictimization. This finding reminds us that, while recognizing that the protective effect of roommate relationships is limited, we should pay attention to the risks that the deterioration of roommate relationships brings to college students’ cybervictimization.

Finally, it is worth noting that although roommate relationships are a kind of interpersonal relationship, they have certain peculiarities compared with general peer relationships. That is, the bonding between members of roommate relationships is often arranged randomly rather than naturally selected, and their daily contact is more frequent and their living space is closer, which also makes roommate relationships more likely to have more complex attributes and mechanisms (Dusselier et al., 2005; Erb et al., 2014). For example, roommate characteristics (ethnicity, life habits, personality traits, etc.), homogeneity among roommates, the structure of the social network in the dormitory and the position of individuals within it, and the type of support that roommates can provide (social comfort, communication, network technology support, etc.), may also have an impact on the role of roommate relationships. However, this study only examined roommate relationships from an overall perspective, which hinders further discussions on the role of roommate relationships. Therefore, future research needs to refine the exploration of roommate relationships’ attributes, dimensions, and types, among others, and further confirm the specific roles that roommate relationships play between cumulative childhood trauma, Internet addiction, and cybervictimization.

### Limitations and implications

This study examined the relationship between Chinese college students’ cumulative childhood trauma and cybervictimization and attempted to explore the mediating role of Internet addiction as a problematic behavior and the moderating role of roommate relationships as an interpersonal factor. A few limitations need to be considered for the study. First, the cross-sectional research design in this study could not obtain continuous data results. In the future, longitudinal research is needed to confirm the model. Second, all the data in this study originated from self-report. Although we used valid items to avoid this problem, childhood trauma and cybervictimization involve negative experiences for individuals, and the results may still be affected by subjective cognition, social approval, and other factors. Therefore, reports from parents and significant others can be considered in future studies. Third, this study only explored roommate relationships as a whole, which has certain limitations in explaining the role of this type of relationship. Future studies need to further control and examine some dormitory-related elements (e.g., cohabitation time, number of roommates, the characteristics of group members), or add mutual evaluation of roommates to conduct a more systematic exploration of the role of roommate relationships. Fourth, the participants of this study were mainly from two universities in southwestern China. More regions should be included in the future. In addition, the investigation time of this study coincided with the COVID-19 pandemic. The impact of isolated dormitory living on Internet addiction and roommate relationships under the pandemic cannot be ruled out. Hence, these results can be reverified in normal times in future.

Despite the above shortcomings, this study has some implications for the prevention of and intervention of cybervictimization. First, the findings suggest that college students who have experienced multiple childhood trauma are also at a higher risk of re-exposure to cybervictimization, suggesting that we should not focus only on the impact of the single adverse experience when intervening with college students in cybervictimization but also on a comprehensive understanding and assessment of individual traumatic experiences. Second, interventions for childhood traumatic experiences and Internet addiction, such as trauma-focused cognitive behavior therapy (Ehring et al., 2014) and Internet addiction-based sports intervention (Liu et al., 2017), can effectively help college students reduce the risk of cybervictimization. Third, as one of the basic interpersonal relationships on Chinese college campuses, roommate relationships deserve more attention. College administrators, teachers, and counseling centers should focus on this issue and help college students with childhood trauma experiences and Internet addiction to acquire skills for dormitory coexistence and communication, coping strategies for dormitory conflict, to name a few. Meanwhile, group sandplay games and group counseling might also be used to prevent or reduce dormitory conflict and thus reduce the risk of cybervictimization.

## Data availability statement

The datasets generated for this study are available on request to the corresponding author.

## Ethics statement

The studies involving human participants were reviewed and approved by the Research Ethics Committee of the School of Education, Soochow University. The participants provided their written informed consent to participate in this study.

## Author contributions

YX designed the study, collected data, and wrote the manuscript. JW obtained funding and reviewed the manuscript content. CZ participated in reviewing the literature and analyzing data. LZ assisted with translation and proofreading of the manuscript. All authors contributed to the development of this manuscript, reviewed drafts, and approved the final version.
